# Lactate dehydrogenase-5 (LDH-5) overexpression in non-small-cell lung cancer tissues is linked to tumour hypoxia, angiogenic factor production and poor prognosis

**DOI:** 10.1038/sj.bjc.6601205

**Published:** 2003-08-26

**Authors:** M I Koukourakis, A Giatromanolaki, E Sivridis, G Bougioukas, V Didilis, K C Gatter, A L Harris

**Affiliations:** 1 Department of Radiotherapy/Oncology, Democritus University of Thrace, Alexandroupolis 68100, Greece; 2 Department of Pathology, Democritus University of Thrace, Alexandroupolis 68100, Greece; 3 Department of Cardiac/Thoracic Surgery, Democritus University of Thrace, Alexandroupolis 68100, Greece; 4 Department of Pathology, Nuffield Department of Clinical Laboratory Sciences, John Radcliffe Hospital, Oxford OX3 9DS, UK; 5 Cancer Research UK, Molecular Oncology Laboratories, Weatheral Institute of Molecular Medicine, Oxford OX3 9DS, UK

**Keywords:** lactate dehydrogenase, HIF, CA9, non-small-cell lung cancer

## Abstract

Lactate dehydrogenase-5 (LDH-5) catalyses the reversible transformation of pyruvate to lactate, having a principal position in the anaerobic cellular metabolism. Induction of LDH-5 occurs during hypoxia and LDH-5 transcription is directly regulated by the hypoxia-inducible factor 1 (HIF1). Serum LDH levels have been correlated with poor prognosis and resistance to chemotherapy and radiotherapy in various neoplastic diseases. The expression, however, of LDH in tumours has never been investigated in the past. In the present study, we established an immunohistochemical method to evaluate the LDH-5 overexpression in tumours, using two novel antibodies raised against the rat muscle LDH-5 and the human LDH-5 (Abcam, UK). The subcellular patterns of expression in cancer cells were mixed nuclear and cytoplasmic. In direct contrast to cancer cells, stromal fibroblasts were reactive for LDH-5 only in a minority of cases. Serum LDH, although positively correlated with, does not reliably reflect the intratumoral LDH-5 status. Lactate dehydrogenase-5 overexpression was directly related to HIF1*α* and 2*α*, but not with the carbonic anhydrase 9 expression. Patients with tumours bearing high LDH-5 expression had a poor prognosis. Tumours with simultaneous LDH-5 and HIF1*α* (or HIF2*α*) overexpression, indicative of a functional HIF pathway, had a particularly aggressive behaviour. It is concluded that overexpression of LDH-5 is a common event in non-small-cell lung cancer, can be easily assessed in paraffin-embedded material and provides important prognostic information, particularly when combined with other endogenous markers of hypoxia and acidity.

Rapid cancer cell proliferation and high metabolic demands, defective structural and functional angiogenesis, as well as irregular spatial relation between cancer cells and stromal vasculature are the principal causes of intratumoral hypoxia (Giatromanolaki and Harris, 2001; Vaupel *et al*, 2001). Reduced oxygen tension is a well-recognised cause of failure of radiotherapy, as reduced intracellular oxygen levels result in decreased free radical formation during irradiation and consequently to a lower amount of DNA strand breaks (Gray *et al*, 1953; Brown, 2002). Furthermore, the molecular cascade triggered in the context of cancer cell response to the hypoxic stress establishes an aggressive phenotype with increased metastatic potential and resistance to various apoptotic stimuli (Rofstad, 2000; Harris, 2002).

Glycolysis is a major source for the production of ATP from glucose, which is transformed to pyruvate while NAD+ is reduced to NADH. The fate of the pyruvate molecules produced depends on the oxygen presence. Adequate oxygenation allows the conversion of pyruvate to ATP, water and carbon dioxide, the reversible transformation of which to carbonic acid is catalysed by the enzyme carbonic anhydrase (Opavsky *et al*, 1996). Lack of oxygen or biochemical defects relevant to the electron transport system or the citric acid cycle restricts ATP production to glycolysis. The glycolytic ATP production will continue as long as NAD+ is available, the availability of which is guaranteed if oxidation of NADH back to NAD+ is feasible. Such an oxidation occurs during the reversible transformation of pyruvate to lactate, a reaction catalysed by lactate dehydrogenase-5 (LDH-5) (Holbrook *et al*, 1975). Lactate dehydrogenase-5 is one of the five isoenzymes of the LDH, composed of four M-subunits and has the highest efficiency among all other isoenzymes to catalyse pyruvate transformation to lactate. The higher the number of H-subunits the LDH contains, the lower the ability of the enzyme to catalyse the reaction, so that LDH-1 (composed of four H-subunits) favours the conversion of pyruvate to acetyl-CoA that enters into the citric acid (Krebs) cycle. Upregulation of LDH-5 by cancer cells guarantees a strong glycolytic metabolism and reduced dependence of cells in the presence of oxygen. The hypoxia-inducible factors 1*α* and 2*α* (HIF*α*s) are key transcription factors regulating glycolysis and the transcription of both LDH-5 and carbonic anhydrase 9 (CA9) (Firth *et al*, 1995; Semenza *et al*, 1996; Ebert and Bunn, 1998; Wykoff *et al*, 2001).

In the present study, we compared the overexpression of LDH-5 and CA9 in non-small-cell lung cancer (NSCLC). Increased activity of LDH, being linked with intratumoral hypoxia and acidity (Yamagata *et al*, 1998), should be related with aggressive tumour features as shown in a previous study on CA9 in NSCLC (Giatromanolaki *et al*, 2001a). As HIF*α*s regulate glycolysis and LDH transcription, we further assessed the HIF association with LDH expression. The prognostic relevance of LDH in comparison with CA9 and HIF*α*s was also assessed.

## MATERIALS AND METHODS

Archival paraffin-embedded biopsy material from 76 primary squamous cell lung carcinomas and 36 lung adenocarcinomas were retrieved, and 3 *μ*m tissue sections were cut on slides (Department of Pathology, University of Oxford, UK). All patients had early operable cancer (T1,2–N0,1 stage) and were treated with surgery alone. Histological diagnosis, grading and N-stage were performed on haematoxylin–eosin-stained sections.

A total of 87 patients were male and 25 female, their ages ranging from 35 to 74 years (median 63). Survival data were available for all 104 patients. At the time of analysis, 54 patients out of 112 were dead, while the median follow-up of surviving patients was 57 months (range 22–83 months).

Cancer tissue samples examined were chosen from the tumour periphery, so that normal lung was included. Specific conditions around the tumour (hypoxia, acidity, high growth factor concentration) could have affected the LDH-5 expression status in the adjacent lung. To avoid such a bias, we further assessed the expression of LDH-5 protein in: (i) 10 tissue samples containing normal alveolar and bronchial tissue obtained from autopsies and (ii) 10 samples from apparently normal lung (located away from the tumour) obtained from patients who underwent pneumonectomy. These samples were retreived from the archives of the department of Pathology, Democritus University of Alexandroupolis, Greece.

### LDH-5 immunohistochemistry

The sheep polycloncal ab9002 (Abcam, Cambridge, UK) raised against human LDH-5 purified from human placenta, and the goat polyclonal ab7639 (Abcam, Cambridge, UK) raised against rabbit muscle lactate dehydrogenase were used for immunohistochemistry (Zaman *et al*, 1999). Ab9002 is an IgG fraction, the identity of which was confirmed by double diffusion against purified LDH-5 and a known anti-human LDH-5. Specificity has been demonstrated by Western blot against liver cell lysate. Ab9002 is available in liquid form, in glycine-buffered saline pH 7.4, 0.1% sodium azide, 0.1% EACA, 0.01% benzamidine and 1 mM EDTA. Ab7639 is an IgG fraction antibody purified from monospecific antiserum by a multistep process including delipidation, salt fractionation and ion exchange chromatography followed by extensive dialysis against a buffer composed of 0.15 M Nacl, 0.01% sodium azide at pH 7.2. Ab7639 is available in liquid form, in protease-free 10 mg ml^−1^ bovin serum albumin BSA IgG and 0.01% gentamicin sulphate.

A modified streptavidin technique was used for immunohistochemistry. Following multiple experiments, the optimal concentration of the Ab standardised for immunohistochemistry in paraffin-embedded tissues was 50 *μ*g ml^−1^ (1 : 200) for both antibodies. Sections were deparaffinised and peroxidase was quenched with methanol and H_2_O_2_ 3% for 15 min. Microwaving for antigen retrieval was used (3 × 5 min). The primary antibody was applied overnight. Following washing with TBS, sections were incubated with a secondary antibody (Kwik Kit, Cat. No. 404050, Thermo Shandon, Pittsburgh, PA 15275, USA) for 15 min and washed in TBS. Kwik streptavidin peroxidase reagent was applied for 15 min and sections were again washed in TBS. The colour was developed by 15 min incubation with DAB solution and sections were weakly counterstained with haematoxylin. Rat renal tissue obtained from hypoxic kidneys after 20 min ligation of the vessels were used for positive controls. Normal goat and sheep immunoglobulin-G was substituted for primary antibody (ab7639 and ab9001, respectively) at a concentration where immunostaining of control slides gave a faint cytoplasmic staining.

### Assessment of HIF*α* and of CA9 expression

The HIF1*α* and HIF2*α* proteins were detected using the ESEE 122 (IgG1 Mab; dilution 1 : 20) and the EP190b (IgG1 Mab; neat) monoclonal antibodies as we previously described (Talks *et al*, 2000). For the detection of CA9, we used the alkaline phosphatase/antialkaline phosphatase (APAAP) procedure and the mouse monoclonal anti-human CA9 antibody M75 (Pastorek *et al*, 1994). The immunohistochemical technique, assessment of expression and identifications of groups of high *vs* low HIF or CA9 reactivity used have been described in previous studies (Giatromanolaki *et al*, 2001a, 2001b). Assessment was based on the percentage of cancer cells with strong cytoplasmic/nuclear HIF*α* reactivity and of membrane CA9 expression.

### Immunohistochemistry for angiogenesis and angiogenic factor expression

The LDH expression was further analysed in comparison with the microvessel density and the expression of angiogenic factors vascular endothelial growth factor (VEGF; VG1 MoAb, University of Oxford, UK), thymidine phosphorylase (TP; PGF-44c, University of Oxford, UK) and basic-Fibroblast growth factor (bFGF) and with the bek-bFGF-receptor (Santa Cruz Biotechnology, USA). The activated VEGF/KDR expressing vascular density was also assessed using the 11B5 Moab (Texas University, USA). Details on the immunohistochemistry and assessment of these parameters have been reported in previous studies of ours (Koukourakis *et al*, 2000; Giatromanolaki *et al*, 2000).

### Comparison of serum LDH *vs* immunohistochemistry

In an additional cohort of 33 patients, the LDH serum levels were prospectively assessed with standard biochemical assays. Serum samples were taken immediately before bronchoscopic biopsy (11 patients with inoperable stage IIIb NSCLC) or on the day of operation (22 patients with stage II/IIIa NSCLC who underwent partial or total pneumonectomy). Lactate dehydrogenase serum levels were further assessed 8 days after biopsy or surgery, respectively. Biopsy or surgical material was formalin-fixed and paraffin-embedded, while LDH immunohistochemistry was performed and assessed, the pathologists being blinded to the results of LDH biochemistry. In this way, we could assess the correlation between serum and tissue LDH and, furthermore, we could study the effect of surgery or biopsy on serum LDH levels. The normal levels of serum LDH in our laboratory are <450 IU l^−1^. Lactate dehydrogenase levels higher than this value were considered as abnormally high.

### Statistical analysis

Statistical analysis and graphs were performed using the GraphPad Prism® 2.01 and the Instat® 3.0 packages (San Diego California USA, www.graphpad.com). The *χ*^2^ *t*-test, Fisher's exact *t*-test or the unpaired two-tailed *t*-test was used for testing relationships between categorical tumour variables, as appropriate. Linear regression analysis was used to test the relationship between continuous variables. Survival curves were plotted using the method of Kaplan and Meier, and the log-rank test was used to determine statistical differences between life tables. A Cox proportional hazard model was used to assess the effects of patient and tumour variables on overall survival. All *P*-values are two sided and *P*-values <0.05 were used for significance.

## RESULTS

### LDH-5 expression patterns

Normal lung cells (bronchial and alveolar) away from the tumour were unreactive for LDH-5, while positive staining was occasionally noted in normal lung adjacent to the tumour. Chondrocytes of entrapped cartilage showed nuclear patterns of expression. The same patterns of staining were obtained with both antibodies used.

**Figure 1:**
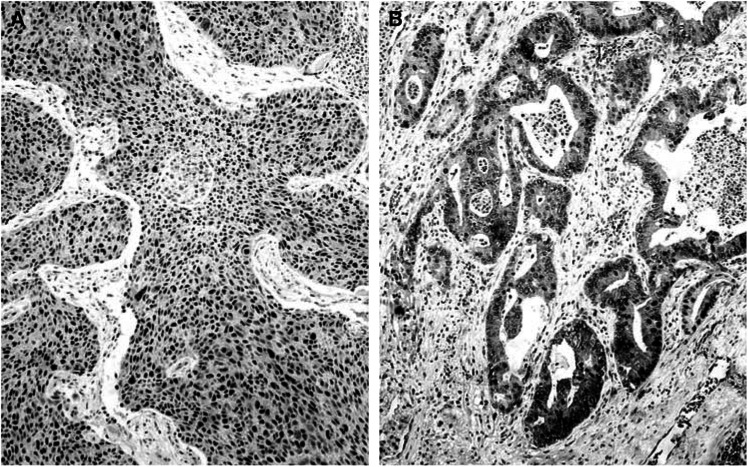
(**A**) A shows a squamous cell lung carcinoma with nuclear and cytoplasmic LDH-5 overexpression and (**B**) a lung adenocarcinoma with predominantly cytoplasmic reactivity.

Immunohistochemical expression of LDH-5 in cancer cells revealed both cytoplasmic and nuclear staining patterns. Nuclear expression was usually accompanied by strong cytoplasmic expression of LDH-5, while predominantly cytoplasmic expression was noted in a subset of tumours (Figure 1a,b

). Linear regression analysis between the percentage of cells with LDH-5 expression obtained with the ab9002 and ab7639 showed a highly significant correlation (*P*<0.0001, *r*=0.92). All subsequent analyses performed were based on the results obtained with the ab9002 (anti-human LDH-5). The median percentage of cells with cytoplasmic LDH-5 expression was 80 (range 0–100; mean 68, s.d. 31). The median percentage of cancer cells with nuclear LDH expression was 10 (0–100; mean 30, s.d. 37). Linear regression analysis revealed a highly significant association between the percentage of cells with cytoplasmic and with nuclear expression (*P*<0.0001, *r*=0.64).

Stroma fibroblasts and vessels were also stained for LDH-5 in 24 out of 112 of the cases analysed, the patterns being predominantly cytoplasmic and to a lesser extent nuclear. In all these 24 cases, extensive cancer cell LDH-5 expression was also present. These staining patterns were obtained with both antibodies used.

### Definition of cutoff points

Cases with cytoplasmic LDH-5 reactivity higher than the median (>80%) were grouped as bearing high LDH-5 reactivity (45 cases). As this cutoff point was very high due to the frequent and abundant LDH-5 overexpression in NSCLC, the remaining cases were divided into two groups of low (0–50%; 47 cases) and medium (51–80%; 30 cases) LDH-5 reactivity.

Using the median percentage of cells with nuclear patterns of LDH expression,two groups of cases with low *vs* high LDH-5 nuclear reactivity were defined (low: 0–10% of cells with nuclear LDH-5 expression, 59 cases; high: >10% of cells with nuclear LDH-5 expression, 53 cases).

### Lactate dehydrogenase association with histopathological variables

No association of cytoplasmic LDH-5 expression with histology (adenocarcinomas *vs* squamous cell carcinomas) or histological differentiation was noted. High cytoplasmic LDH-5 expression was more frequent in advanced T-stage (*P*=0.10) and nodal involvement (*P*=0.15), but the difference was not statistically significant. Expression of LDH-5 was independent of the degree of necrosis. Similarly, nuclear patterns of LDH-5 expression did not relate to any of the histopathological variables examined.

### Lactate dehydrogenase association with HIF*α*s and CA9

Linear regression analysis between the percentage of cells with cytoplasmic LDH-5 expression and the percentage of cells positive for HIF1*α* and HIF2*α* revealed a significant positive correlation (*P*<0.0001, *r*^2^=0.41 and *P*=0.03, *r*^2^=0.20, respectively). No association with CA9 expression was noted (*P*=0.72, *r*=0.03). Similar analysis using the percentage of cells with nuclear LDH reactivity did not show any association.

Using categorical variable analysis, high cytoplasmic and high nuclear LDH-5 expression were significantly linked with high HIF1*α* expression, but not with HIF2*α* or CA9 expression (Table 1

**Table 1:** Association of LDH cytoplasmic and nuclear expression with HIF1*α*, HIF2*α* and CA9 expression

	**LDH cytoplasmic**				**LDH nuclear**		
	**Low**	**Medium**	**High**	***P*-value**	**Low**	**High**	***P*-value**
HIF1a							
Low	24	10	8	<0.0001	28	14	0.03
high	13	20	37		31	39	
							
HIF2a							
Low	22	14	18	0.21	31	23	0.35
high	15	16	27		28	30	
							
CA9							
Low	22	17	24	0.85	35	28	0.56
high	15	13	21		24	25	

).

### Lactate dehydrogenase association with angiogenesis

Using LDH-5 and all angiogenic parameters as continuous variables, linear regression analysis showed a significant association of cytoplasmic (but not of nuclear) LDH-5 with most of the angiogenic factors, but not with microvessel density (Table 2

**Table 2:** Linear regression analysis of LDH cytoplasmic expression with microvessel density and angiogenic factor and receptors

	***P*-value**	***r*-value**
MVD	0.65	0.04
aMVD	0.23	0.12
VEGF	0.01	0.22
bFGF	0.0002	0.35
bFGFr	0.01	0.23
TP	0.04	0.20

). Categorical variable analysis (using groups as defined in previously reported studies (Koukourakis *et al*, 2000; Giatromanolaki *et al*, 2000) showed similar results (data not shown). Briefly, a significant association of high LDH-5 cytoplasmic expression with VEGF cytoplasmic expression was noted (*P*=0.01). Similarly, a significant association of LDH-5 cytoplasmic expression with bFGF and bek-bFGF-receptor expression was confirmed (*P*=0.004 and 0.05, respectively), while the association with TP expression was marginal (*P*=0.07). The association of LDH-5 nuclear expression with these variables was less strong and did not reach significance. Lactate dehydrogenase-5 expression was not associated with microvessel density or the activated VEGF/KDR microvessel density (aMVD).

### Association of HIF*α*s with VEGF and CA9

The association among these variables in NSCLCs, in the same material used in the present study, has been extensively analysed in two previous studies (Giatromanolaki *et al*, 2001a, 2001b). Briefly, VEGF is directly related to HIF1*α* and HIF2*α* but not with CA9. A marginal, not significant, association between CA9 expression and HIF1*α* was also noted (data not shown).

### LDH-5 and overall survival

Figure 2

**Figure 2:**
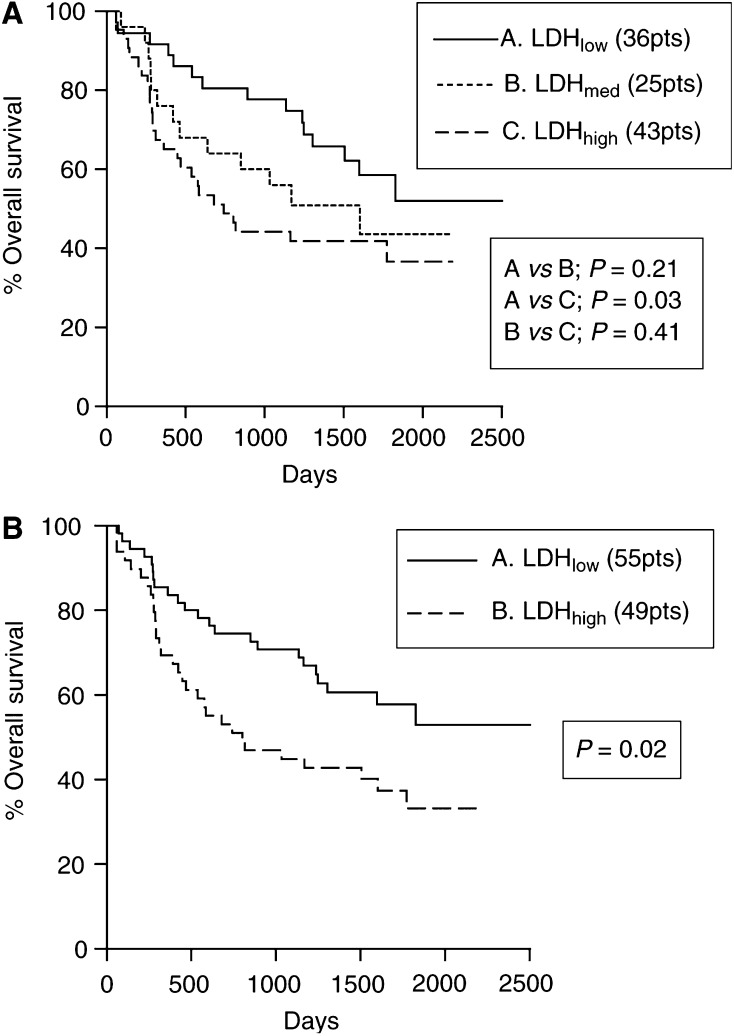
Kaplan–Meier overall survival curves stratified for cytoplasmic (**A**) and nuclear (**B**) LDH-5 reactivity.

shows the Kaplan–Meier overall survival curves stratified for cytoplasmic LDH-5 (low *vs* medium *vs* high) and nuclear (low *vs* high) LDH-5 expression. A significantly poorer survival was noted in the group of patients with high LDH-5 cytoplasmic and high LDH-5 nuclear reactivity (*P*=0.03 and 0.02, respectively).

**Figure 3:**
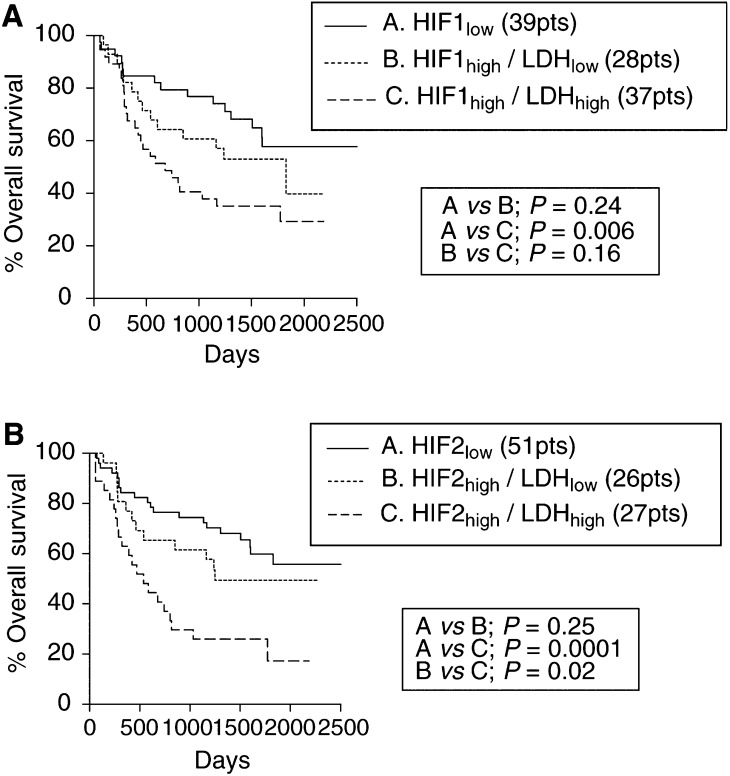
Kaplan–Meier overall survival curves following double stratification for nuclear LDH-5 reactivity and HIF1a (**A**) or HIF2a (**B**) overexpression.

In multivariate analysis using a model that comprised the T-, N-stage, CA9 and HIF*α*s expression, LDH-5 expression did not reach an independent prognostic significance (*P*=0.15, *t*-ratio=1.44), probably due to the close association with HIF*α*s and angiogenic factor expression. However, the prognostic usefulness of LDH-5 as a marker is better revealed in double stratification with HIF*α*s. Overexpression of LDH-5 is a marker of a functional HIF*α* pathway, and as such LDH-5 could enhance the prognostic usefulness of HIF*α*s. Indeed, HIF1*α* and 2*α* overexpression related to poor overall survival only when LDH-5 was also overexpressed (whether cytoplasmic or nuclear). Figures 3a and b

show the Kaplan–Meier survival curves according to HIF1*α* and 2*α* expression, respectively, stratified for nuclear LDH-5 reactivity. In multivariate analysis, the combination of nuclear LDH-5 and HIF2*α* expression showed a very strong independent prognostic relevance. Table 3

**Table 3:** Multivariate analysis of the impact of combined expression of HIFás and the nuclear expression of LDH-5 on death events in three statistical models

	**Model I**		**Model II**		**Model III**	
**Parameter**	***t*-ratio**	***P*-value**	***t*-ratio**	***P*-value**	***t*-ratio**	***P*-value**
HIF1*α*/LDH-5	0.46	0.64	—	—	—	—
HIF2*α*/LDH-5	2.53	0.01	2.79	0.006	3.02	0.003
CA9	1.03	0.30	1.07	0.28	—	—
N-stage	2.93	0.004	2.90	0.004	3.01	0.003
T-stage	1.86	0.06	1.81	0.07	2.54	0.01
Grade	0.43	0.66	0.37	0.70	—	—
Histology	1.13	0.26	1.20	0.23	—	—
VEGF	0.17	0.85	0.15	0.87	—	—

shows the multivariate analysis in three statistical models and the relative risk.

**Figure 4:**
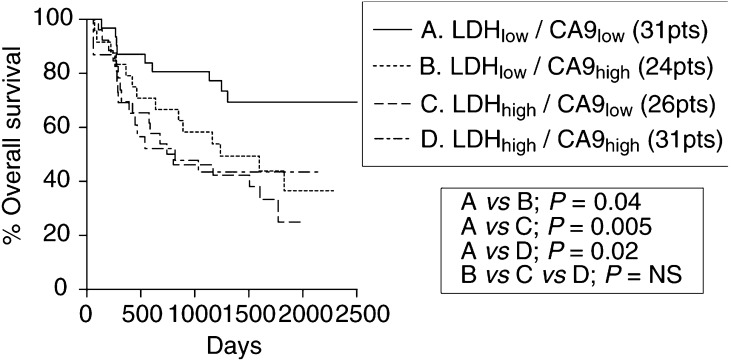
Kaplan–Meier overall survival curves stratified for nuclear LDH-5 and membrane CA9 reactivity.

An additional survival analysis was performed by stratifying for LDH-5 nuclear and CA9 overexpression. Again, upregulation of either of the enzymes was linked with poor survival (Figure 4

).

### Serum LDH levels *vs* immunohistochemistry

**Figure 5:**
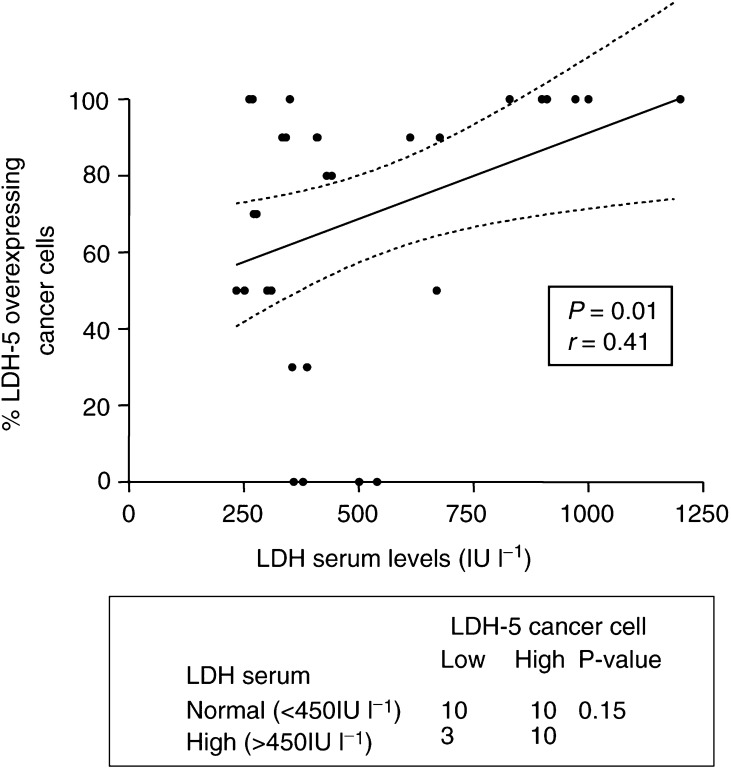
Correlation between serum and tissue levels of LDH-5.

The median value of LDH serum levels was 410 IU l^−1^ (range 234–1294). In all, 20 out of 33 patients had LDH serum levels lower than the upper normal LDH value (450 IU l^−1^), while in 13 the LDH serum levels were higher than the normal. Linear regression analysis between the serum LDH levels and the percentage of cancer cells with cytoplasmic (and/or nuclear) LDH-5 reactivity showed a statistically significant correlation (*P*=0.01, *r*=0.41; Figure 5

). Categorical analysis showed, however, that the results from the two methods are not overlapping as 10 out of 20 patients with high LDH-5 tissue reactivity had LDH serum levels lower than the highest normal value (Figure 5). The tumour stroma LDH-5 immunohistochemical reactivity was not related with serum LDH levels (data not shown).

In accordance with the previously reported results, in this series of 33 patients a significant association of tissue LDH-5 expression with HIF1*α* was recorded, while serum LDH levels were not significantly related to HIF1*α* overexpression (data not shown).

**Figure 6:**
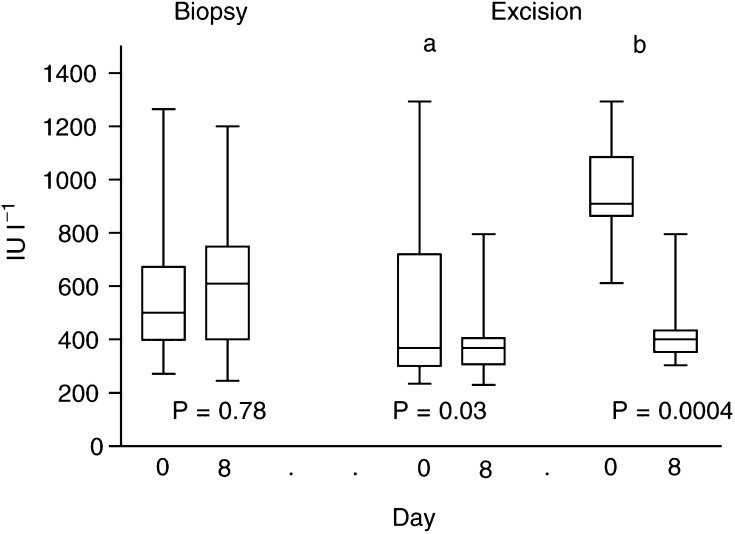
Lactate dehydrogenase serum levels before and after biopsy or surgery, in patients with non-small-cell lung cancer. Group ‘a’ refers to all patients and group ‘b’ to patients with preoperative high LDH levels.

At eight days following biopsy, the mean serum LDH levels were unchanged (592±300 *vs* 636±317; *P*=0.78), while a statistically significant drop was noted in patients who underwent surgery (527±329 *vs* 369±117; *P*=0.03). The sharp drop of LDH serum levels was more evident in patients with high preoperative LDH serum levels (*P*=0.0004); Figure 6

.

## DISCUSSION

Lactate dehydrogenase, assessed in the sera of cancer patients, is considered as a marker of poor postoperative outcome in a large spectrum of neoplastic diseases including pancreatic carcinoma (Tas *et al*, 2001a), osteosarcoma (Ferrari *et al*, 2001), renal and testicular carcinoma (Motzer *et al*, 1999; vonEyben *et al*, 2001) and melanoma (Hauschild *et al*, 1999). An increased metastatic potential of nasopharyngeal carcinomas in patients with high LDH serum levels has also been reported (Cheng *et al*, 1998). Furthermore, clinicopathological studies suggest that high LDH serum levels are linked with radioresistance of head–neck cancer (Brizel *et al*, 2001), brain primary and metastatic tumours (Kushner *et al*, 2001; Lutterbach *et al*, 1999; Lagerwaard *et al*, 1999). Elevated LDH serum levels are also linked with resistance to and high rate of relapse after chemotherapy in lymphomas (Kondo *et al*, 2001; Wilder *et al*, 2001), breast and ovarian cancer (Ryberg *et al*, 2001; Yuce *et al*, 2001), as well as in small cell lung cancer (Argiris *et al*, 2001; Tas *et al*, 2001b).

The ominous prognostic significance of serum LDH in malignancy could be attributed to several reasons: (i) increased LDH activity results in lactic acid production and acidification of the extracellular water space, which is a very common feature in cancer (Vaupel *et al*, 1989; Stubbs *et al*, 2000). Acidic extracellular pH has been shown to activate gelatinase activity and production of cathepsin D, which contribute to an increased cancer cell invasive ability (Rozhin *et al*, 1994; Martinez-Zagulian *et al*, 1996). Activation of macrophage-mediated angiogenesis by lactate may also facilitate metastasis (Jensen *et al*, 1986; Zabel *et al*, 1996; Murray and Wilson, 2001). Moreover, low pH protects mitochondria from oxidative stress and may account for increased resistance of cancer cells to hypoxia-induced apoptosis (Bronk and Gores, 1991; Nemoto *et al*, 2000); (ii) increased LDH production by cancer cells can be a direct marker of intratumoral hypoxia (Firth *et al*, 1995), and therefore a strong marker of tumour resistance to radiotherapy and some chemotherapeutic agents; (iii) as LDH-5 is transcriptionally regulated by HIF*α*s, high LDH serum levels may reflect an upregulated HIF-molecular cascade (Firth *et al*, 1995; Semenza *et al*, 1996; Ebert and Bunn, 1998). Hypoxia inducible factor stabilisation that occurs due to hypoxia or genetic mutations results in the overexpression of a variety of proteins linked to angiogenesis/metastasis, glycolysis and resistance to apoptosis (Semenza, 1998; Akakura *et al*, 2001; Harris, 2002). In that way, high serum LDH levels may be an indirect marker of HIF-dependent tumour aggressiveness and resistance to cytotoxic regimens (Aebersold *et al*, 2001; Koukourakis *et al*, 2001b; Koukourakis *et al*, 2002).

Serum levels, however, are unlikely to reflect adequately the intratumoral LDH activity, as the bulk of the neoplastic disease or individual variations of LDH clearance are strong confounding factors. On the other hand, biochemical assessment of LDH from tumour tissue extracts (Beasley *et al*, 2000) give measurements that strongly vary with the amount of the nonmalignant cellular component (stromal and reactive cells) or even with the extent of necrotic tissue that composes the tumour sample used to obtain tissue extracts. In the present study, we showed that immunohistochemical assessment of LDH-5 allows (i) the differential assessment of LDH-5 production by cancer and noncancerous cells of the tumours, (ii) the intensity of LDH-5 activity within cancer cells and (iii) the identification of subcellular patterns of LDH-5 localisation that could be of biological relevance. Immunohistochemistry, therefore, allows the characterisation of the individual cancer cell LDH activity, which cannot be adequately predicted by serum LDH levels or tissue LDH biochemistry. Indeed, comparative analysis of serum LDH levels and immunohistochemistry showed a positive trend and the results between the two methods were not overlapping.

The patterns of LDH-5 subcellular expression by cancer cells were cytoplasmic with a varying degree of nuclear localisation. Lactate dehydrogenase is well known to reside in both the cytoplasm and nuclei (Reddy and Shukla, 2001). In the majority of cases, the tumoral stroma (fibroblasts, vessels and reactive cells) was unreactive for LDH-5, while in a minority of cases extensive cytoplasmic expression of LDH-5 in stromal cells accompanied LDH-5 overexpression by cancer cells. This finding, together with the observation that serum LDH levels correlated positively with cancer cell LDH-5 reactivity but not with stroma reactivity, strongly suggests that high LDH levels found in the sera of cancer patients are mainly of cancer cell origin, while the contribution of stroma cells is minimal. The rapid drop of LDH serum levels 8 days following surgery further confirms that the excess serum LDH levels found in a subset of NSCLC patients is of tumour origin.

We further assessed whether the LDH-5 expression status by cancer cells relates to any of the histological tumour characteristics. In a previous study by Mall *et al* (2002), serum LDH levels correlated with advanced stage in small cell lung cancer, and similar findings have been reported for ovarian cancer (Yuce *et al*, 2001). Poor differentiation has also been linked with high LDH levels in squamous cell HNC (Ross *et al*, 2000). An increased incidence of metastasis in patients with Ewing's sarcoma or nasopharyngeal carcinoma has also been reported to relate with high serum LDH levels (Cheng *et al*, 1998; Bacci *et al*, 2000). In our study, overexpression of LDH-5 by cancer cells was more frequent in large tumours or in cases with node involvement, but this was not statistically significant. Tumour histological type and differentiation or the extent of necrosis were not related to LDH-5 expression.

A strong significant association of LDH-5 overexpression with HIF1*α* and to a lesser extent with HIF2*α* was noted, which is in full accordance with studies showing that LDH-5 is transcriptionally regulated by the HIF*α*s (Semenza *et al*, 1996; Ebert and Bunn, 1998). Lactate dehydrogenase-5 expression patterns were similar to that observed in HIF*α* immunostaining, in that reactivity when present was diffuse and not around necrotic areas (Giatromanolaki *et al*, 2001b). This is in direct contrast with the expression of CA9, where its expression was strongly related with the extent of necrosis and most often localised in cancer cell layers proximal to necrotic areas (Giatromanolaki *et al*, 2001a). Lactate dehydrogenase-5 was not linked with CA9 expression. Although CA9 gene expression is HIF*α* regulated (Wykoff *et al*, 2001), the threshold of hypoxia necessary for the transcription of CA9 or LDH-5 may differ (Giatromanolaki *et al*, 2001a), which could explain the lack of association between LDH and CA9 expression as well as the discordant relation of these proteins with necrosis. Nevertheless, HIF*α*s and LDH-5 are additionally regulated by oncogenes and cytokines, which may account for their more diffuse expression than CA9.

In some tumours, overexpression of HIF1*α* was not accompanied by LDH-5 overexpression. This could show a defective HIF1*α* pathway, or tumour and gene polymorphism differences in the regulation of individual genes. Alterations on the expression or function of molecules involved in the HIF-DNA binding may exist (Ebert and Bunn, 1998; Ema *et al*, 1999). On the other hand, the overexpression of LDH in a minority of cases with no HIF*α* reactivity may indicate hypoxia independent pathways of LDH-5 transcriptional regulation, for example, c-myc activation (Shim *et al*, 1997).

Combined assessment of HIF1*α* with LDH-5 expression could therefore predict for an intact or defective HIF pathway. This hypothesis was tested in the survival analysis performed in the present study. In a previous study we showed that HIF*α*s overexpression is linked with poor postoperative outcome in NSCLC. In the present study LDH-5 overexpression was similarly linked with poor prognosis. Double stratification analysis showed that HIF1*α* and HIF2*α* overexpression defined poor prognosis only when LDH-5 was overexpressed. This observation suggests that combined HIF*α* and LDH-5 assessment is a more potent prognostic tool, reflecting the downstream programme regulated by HIF as being a profile mediating poor prognosis. Further analysis of LDH-5 together with CA9 expression revealed that overexpression of either of the enzymes is independently linked with poor survival. Acidification of the tumour environment expected in both conditions explains in part such an aggressive tumour behaviour (Rozhin *et al*, 1994; Martinez-Zagulian *et al*, 1996; Stubbs *et al*, 2000).

We further examined the association of LDH-5 expression with angiogenesis and activation of various angiogenic pathways. Although LDH was not related to the microvessel density, a significant association of LDH-5 with VEGF, bFGF and to TP expression was noted. Vascular endothelial growth factor coexpression with LDH was expected not only due to the HIF*α* dependent regulation of both molecules, but also due to a direct effect of acidosis on VEGF upregulation (Fukumura *et al*, 2001; Shi *et al*, 2001). In two previous studies, using the same material herein analysed, we showed that VEGF is directly associated with the expression of HIF1*α* and HIF2*α*, while CA9 expression was marginally associated with HIF1*α* (Giatromanolaki *et al*, 2001b, c). A significant association between LDH and VEGF levels in the pleural fluid of patients with various diseases has been reported (Cheng *et al*, 1999). The strong association of LDH with bFGF activated pathway, however, may be due to HIF-independent reasons. For example, acidification of the tumour environment by lactate may account for bFGF overexpression (D'Arcangelo *et al*, 2000). On the other hand, a direct effect of bFGF on LDH cellular levels has been recently shown by Riera *et al* (2002).

The present study provides the basis for the immunohistochemical assessment of LDH in tumoral tissues. Lactate dehydrogenase immunohistochemistry can better predict the cancer cell LDH activity than serum LDH or biochemistry on tissue extracts. Lactate dehydrogenase cancer cell overexpression related to HIF*α* overexpression, and the abundant expression of various angiogenic factors. While LDH cancer cell expression represents an important tool to predict aggressive tumour behavior, it becomes a more powerful prognostic tool when combined with HIF*α* expression. Downregulated LDH on the background of HIF*α* overexpression allows the identification of a subgroup of tumours with a less aggressive pattern of gene expression regulated by HIF. This may prove to be of importance as therapeutic strategies targeting HIF activity become available (Kung *et al*, 2000). As endogenous markers of hypoxia have recently focused attention as predictors of response to radiotherapy and chemotherapy (Aebersold *et al*, 2001; Koukourakis *et al*, 2001a, 2001b, 2002), combined HIF/LDH analysis may become of value. The expression of LDH was independent of another enzyme involved in tumour acidity and hypoxia, namely CA9, although expression of either of the enzymes related to an ominous prognosis. It seems that LDH, CA9 and HIF*α*s immunohistochemistry can reliably predict tumour aggressiveness related to acidity and hypoxia, although this requires further investigation.
